# An experimental investigation of the combustion performance of human faeces

**DOI:** 10.1016/j.fuel.2016.07.077

**Published:** 2016-11-15

**Authors:** Tosin Onabanjo, Athanasios J. Kolios, Kumar Patchigolla, Stuart T. Wagland, Beatriz Fidalgo, Nelia Jurado, Dawid P. Hanak, Vasilije Manovic, Alison Parker, Ewan McAdam, Leon Williams, Sean Tyrrel, Elise Cartmell

**Affiliations:** Cranfield University, Cranfield, Bedfordshire MK43 0AL, United Kingdom

**Keywords:** Faecal biomass, Combustion, Smouldering, Non-sewered sanitary systems, Nano membrane toilet

## Abstract

Poor sanitation is one of the major hindrances to the global sustainable development goals. The Reinvent the Toilet Challenge of the Bill and Melinda Gates Foundation is set to develop affordable, next-generation sanitary systems that can ensure safe treatment and wide accessibility without compromise on sustainable use of natural resources and the environment. Energy recovery from human excreta is likely to be a cornerstone of future sustainable sanitary systems. Faeces combustion was investigated using a bench-scale downdraft combustor test rig, alongside with wood biomass and simulant faeces. Parameters such as air flow rate, fuel pellet size, bed height, and fuel ignition mode were varied to establish the combustion operating range of the test rig and the optimum conditions for converting the faecal biomass to energy. The experimental results show that the dry human faeces had a higher energy content (∼25 MJ/kg) than wood biomass. At equivalence ratio between 0.86 and 1.12, the combustion temperature and fuel burn rate ranged from 431 to 558 °C and 1.53 to 2.30 g/min respectively. Preliminary results for the simulant faeces show that a minimum combustion bed temperature of 600 ± 10 °C can handle faeces up to 60 wt.% moisture at optimum air-to-fuel ratio. Further investigation is required to establish the appropriate trade-off limits for drying and energy recovery, considering different stool types, moisture content and drying characteristics. This is important for the design and further development of a self-sustained energy conversion and recovery systems for the NMT and similar sanitary solutions.

## Highlights

• Dry human faeces have a Higher Heating Value (HHV) of 24 MJ/kg.
• Faeces combustion was investigated using a bench-scale downdraft combustor test rig.
• Combustion temperature of 431–558 °C was achieved at air flow rate of 10–18 L/min.
• Fuel burn rate of 1.5–2.3 g/min was achieved at air flow rate of 10–18 L/min.
• Combustion temperature of up to 600 ± 10 °C can handle 60 wt.% moisture in faeces.

## 1 Introduction

Human faeces is a rich source of biomass having a mixture of undigested fats, protein, water, polysaccharide, bacterial biomass, gut secretions, cell shedding and ash [1]. This useful resource is typically treated as a waste material, and openly disposed in the environment by the nearly 1 billion people world-wide who have no access or do not use a toilet. It is estimated that 40% of the world’s population (about 2.4 billion people) lack adequate sanitation facilities, particularly in developing countries [2]. In these areas, >90% of the faeces generated are disposed into the open without treatment, polluting surrounding lakes and rivers [3]. Even in communities with modern sanitary systems, wastewater often leak into the environment, due to improper usage and maintenance of septic systems, putting the groundwater at risk of contamination, especially areas with high water table. These scenarios pose a number of health and environmental hazards including the outbreak of infectious diseases and parasitic worms. The world’s population is projected to reach close to 10 billion by 2050 [4]; consequently, poor sanitation is projected to increase due to rapid urbanization and overburdening of already stressed waste treatment systems. Therefore, the development and universal access of improved sanitation facilities have become a global priority.

The Reinvent the Toilet Challenge (RTTC) of the Bill and Melinda Gates Foundation is set to develop affordable, next-generation sanitary systems that can work without connection to external water, energy or sewerage systems [5]. The Nano-Membrane toilet (NMT) is an example of such a unique off-grid, household-scale toilet solution that is being developed at Cranfield University to safely treat human waste onsite [6]. This unit will integrate a compact energy conversion system that can thermally-treat human faeces, without external energy and water supply. The energy recovered can then be used to meet the toilet’s energy requirement to ensure the self-sustained operation of the NMT unit.

Among thermochemical conversion processes, combustion is a promising and the most mature technology targeted for treating biomass, [7]. It is an exothermic reaction that ensures the complete conversion of a fuel in the presence of an oxidant and heat, with the product gas largely constituted by carbon dioxide (CO_2_). Combustion can consist of rapid oxidation of the fuel, which is characterised by high temperatures of >1000 °C with the visible presence of flames, or a slow, progressive, flameless, and relatively low temperature reaction, referred to as smouldering. The latter involves the oxidation of the fuel in the gas phase surrounding the fuel [8] with progressive burn, spread and heat release rate. Peak temperatures for smouldering are reported in the range of 500–700 °C [8], which could be low as 250 °C [9]. It involves the thermal degradation of the fuel, evolution of volatiles, and resulting visible glow of heat that propagates into flames, depending on oxygen availability, the presence of stable source of heat (external or previously heated material) as well as feedstock composition and characteristics [10], [11].

Smouldering has recently gained importance in practice as an applicable technology. Some of its recent applications include remediation of coal tar [12]; combustion of wood biomass [10], [11], [13]; treatment of bio-solids from wastewater treatment plant [14]; remediation of oil contaminated soil [15] and faeces treatment [16], [17]. Yermán et al. [16] showed that a self-sustained smouldering combustion can be applied to treat moist faeces under given sand pack height, sand-to-faeces ratio, air-to-fuel ratio, and faecal moisture limit. Their studies examined the smouldering combustion of surrogate faeces and validated the process for faeces treatment using dog faeces. Wall et al. [17] investigated the influence of moisture content on smouldering velocity and upscaling of the process. Both studies showed that smouldering can be applied for faeces treatment in on-site sanitation systems, although, this was not demonstrated with human faeces. The operational parameters such as sand pack height and sand-to-faeces mass ratio require sand as a porous medium in the combustor, which are not typical characteristics of a conventional combustor. Thus, little is known about the fundamental combustion processes of human faeces.

Other experimental studies have explored processes such as pyrolysis [18] and hydrothermal carbonization [19] to treat human faeces; although, the development of these technologies is in the early stages and could have added complexities and costs. Ward et al. [18] examined the HHV of real and synthetic faecal char at pyrolysis temperature of 300–750 °C and showed that the faecal char obtained at pyrolysis temperature of 300 °C had a comparable HHV with wood char. Afolabi et al. [19] employed microwave-assisted hydrothermal carbonization to treat human faeces at temperatures of 160–200 °C and residence time of 30–120 min under autogenous pressure, and recovered char and ammonia. These studies confirm that human faeces have unique resource recovery potentials. Monhol and Martins [20] investigated the co-combustion of polyethylene waste and charcoal in a combustion cell and compared the outcomes with the combustion of human faeces. Their study showed that the temperature profile from human faeces combustion was more uniform than co-combustion of polyethylene and charcoal. Earlier studies by the same authors [21] focussed on the ignition properties of these fuels; however, little is shown on the operating conditions for handling the combustion of human faeces.

Unlike well-established fuels with uniform fuel characteristics such as coal, the physical and chemical characteristics of human faeces vary with nutritional intake, health status, gender, body weight and age of individuals [1]. Faeces also possess complex compositional characteristics, such as the presence of a viscous “sticky” substance, possibly from the linings of the intestinal wall, which makes handling and pre-treating of the samples difficult. There is therefore the need to understand the combustion processes of human faeces and establish the right operating range for fuel conversion, considering sample variabilities and uncertainties. This study describes the combustion performance of a bench-scale downdraft combustor test rig when utilised for human faeces combustion. Initial set of analyses were conducted using simulant faeces and wood biomass to ensure repeatability, for fuel comparison and to understand the combustion operating conditions of the test rig. Parameters such as fuel moisture content, air flow rate, fuel pellet size and ignition mode were investigated. Performance evaluation was carried out on the basis of combustion temperature, fuel burn rate, modified combustion efficiency (MCE) and carbon conversion efficiency (η_CCE_).

## 2 Methods

### 2.1 Fuel characterization

About 3 kg of fresh human faeces was collected and stored in a freezer at −85 °C, over a period of two weeks to preserve and prevent microbial degradation of the samples. The frozen samples were thawed at room temperature and mixed until a uniform consistency was obtained. The homogenised human faeces (HF) sample was dried at 45 ± 5 °C in a GENLAB Hot Air Oven to constant weight. The limited drying temperature was applied to prevent the loss of volatile matter. Simulant faeces (SF) were prepared using the recipe outlined in Table 1 [22], while wood biomass (WB) was sourced locally. The relative percentages of carbon (C), hydrogen (H) and nitrogen (N) in the samples were determined using a thermal elemental analyser (Vario ELIII CHN) according to BS EN ISO 16948. The moisture content of the sample was determined at 105 ± 5 °C using the protocol outlined in BS EN 14774-3. The volatile matter and ash content were determined using a Carbolite muffle furnace with the heating conditions in BS EN 15148 and BS EN 14775 respectively. The oxygen (O) content was obtained by subtracting the wt.% percent of C, H, N and ash from 100% while the fixed carbon content was obtained by subtracting the wt.% percent of moisture, ash and volatile matter from 100% [23]. Samples were prepared in triplicates, and mean values are reported for each analysis in Table 2. The HHVs for these samples were determined using Parr Bomb Calorimeter [6400 Automatic Isoperibol].

### 2.2 Experimental set-up

**Fig. 1:**
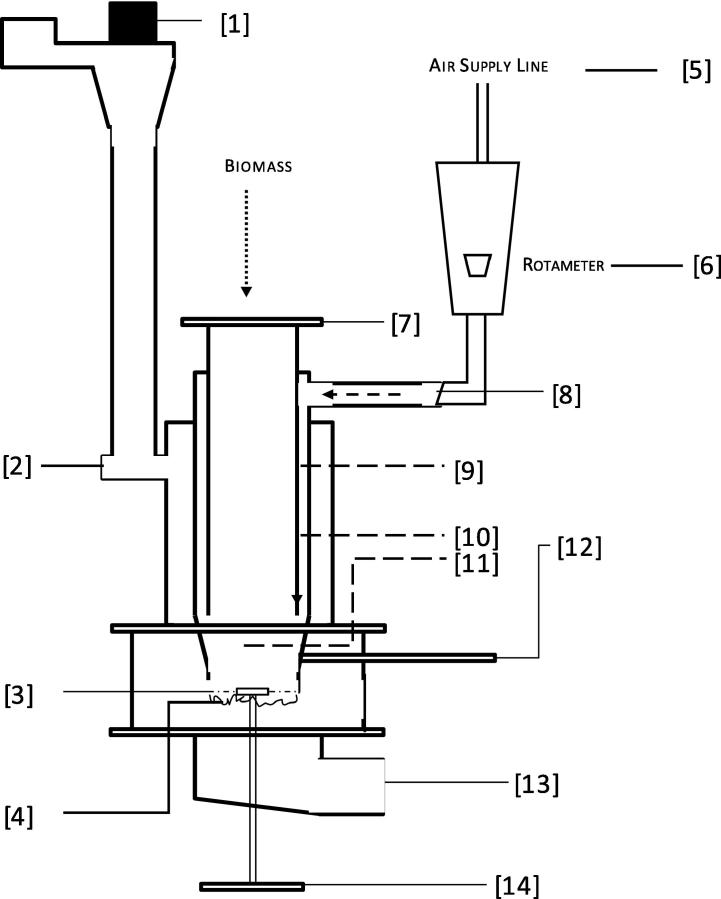
Schematic diagram of the bench-scale fixed bed downdraft combustor test rig. [1] Suction Fan, [2] Exhaust Port, [3] Ash Agitator, [4] Fuel Bed (Grated Surface), [5] Air Supply Line, [6] Rotameter, [7] Fuel Inlet Gate, [8] Primary Air Inlet, [9] Upper Combustor Temperature, [10] Lower Combustor Temperature, [11] Combustion (Bed) Temperature, [12] Heater Temperature/Air Igniter, [13] Ash Collector and [14] Ash Rotor.

The schematic diagram of the experimental test rig used in this study is shown in Fig. 1. This bench scale test rig was provided by RTI International/Colorado State University, as part of the Nano-membrane Toilet Phase II project of the RTTC with the objective to investigate the thermochemical conversion processes of human faeces. The test rig consists of a downdraft reactor with air supply, igniter, fuel grate, fuel feed-in and an ash collection system. The igniter is powered by an AC bench power supply. The reactor is a stainless-steel cylindrical tube with a diameter of 0.07 m and a height of 0.23 m from the fuel inlet gate to the fuel grate.

The biomass material is fed via the fuel inlet gate opening into the reactor (Item 7 in Fig. 1). The fuel, which settles on the fuel bed with grated surface (4), is heated by convective heat transfer from preheated air supply of an AC powered air igniter (12). The heat transfer increases the temperature of the fuel and initiates fuel ignition (standard ignition); aiding the stages of drying, devolatilisation, and subsequent conversion of the fuel to product gas and ash. After fuel ignition, which is evident by a sudden increase in the combustor temperature, the igniter and the preheated air supply are shut off and air is supplied at ambient conditions via the primary air inlet (8). The air from the primary air inlet enters the reactor just about the oxidation zone and moves downward towards the fuel bed. A rotameter (6) is fitted at the primary air inlet (3) to measure the volumetric air flow rate into the reactor. This air stream maintains the release of volatiles from the fuel and the conversion of the formed char into non-condensable gas and ash. Depending on the air flow, incomplete combustion may occur and the product gas may also include a combination of hydrogen (H_2_), carbon monoxide (CO), and methane (CH_4_). Nitrogen (N_2_) from the air is also present in the outlet gas stream. The flue gas leaves the reactor through the exhaust port positioned at the top-side of the unit (9), where it is directed to an extraction hood via a suction fan (1). The suction fan is only operated during fuel ignition as it facilitates the flow of preheated air into the combustion zone by drawing ambient air over the heated igniter surface. The ash that is deposited during combustion is emptied into an ash collector (14). This process is assisted by a small fan-type motor (15) and a metal scraper that rubs over the grate (3). Temperature measurements are achieved with K-type thermocouples positioned at different zones (2, 9–12).

### 2.3 Experimental procedures

For each experiment, 50 g of dry biomass sample (*except stated otherwise*) was introduced into the reactor. This initial amount of fuel was estimated from the maximum adult faeces generation rate of 170 g/cap/day and considering a 25% total solids in human faeces, as reported in [1]. Two fuel ignition modes were tested to understand the best approach for igniting the faecal biomass: (a) standard ignition as explained above, and (b) ‘booster’ ignition, considering the requirement of limited use of energy. For the booster ignition, the suction fan was operated only when the igniter temperature reaches about 600 °C. Similar to the standard ignition procedure, once the fuel is ignited the igniter is switched off and the primary air flow rate is set to the required value. All experiments were carried out feeding the primary air at ambient temperature and pressures. Primary air flow rate ranges from 10 to 18 L/min depending on the experiment. The volumetric compositions of CO, CO_2_, H_2_, and CH_4_ were measured at every second interval and recorded via a data logging system. The gas sampling unit consists of three PTFE filtration and condensation units, connected in series to the gas analyser that is based on thermal conductivity detection. The analyser was calibrated at intervals with a standard gas once a day and at the start of experiment. At the end of the experiment, the leftover ash was collected and weighed. The residual carbon content in the leftover ash is then determined using loss-on-ignition (LOI) approach [24]. The ash blade wiper was not operated during the experimental runs in order to avoid disrupting the reactions occurring in the combustion zone.

### 2.4 Performance evaluation

The operational boundaries and the optimum conditions for converting the faecal biomass were established by varying the: (i) air flow rate, (ii) ignition mode, (iii) fuel pellet size, (iv) bed height, and (v) fuel moisture content. Performance of the process was addressed by determining the MCE and η_CCE_.

The mass flow rate of the primary air (in g/s) was calculated by means of Eq. (1). The flue gas molar flow rate, *x_fg_* was obtained from a mass balance of nitrogen and using the concentration of the constituents in the flue gas. This is expressed in Eq. (2) as g mol gas per second.

(1) $$\text{Air flow rate,AFR}(\text{g}/\text{s})=X_{i}\cdot \rho /60$$

(2) $$x_{\mathrm{fg}}(\text{mol}/\text{s})=\text{AFR}\cdot {\text{V}}_{{\text{N}}_{2}\text{air}}\cdot 100/{\text{M}}_{{\text{N}}_{2}}\cdot \left(100-[{\text{CO}}_{2}\text{,}\%]+[\text{CO,}\%]+[{\text{CH}}_{4}\text{,}\%]+[{\text{H}}_{2}\text{,}\%]\right)$$

where $X_{i}$ is the air volumetric flow rate in L/min as measured by the rotameter, ρ is the density of air, 1.225 g/L at standard temperature and pressure, ${\text{V}}_{{\text{N}}_{2}\text{air}}$ is the mass percentage of nitrogen in air, 76.9%. ${\text{M}}_{{\text{N}}_{2}}$ is the molecular weight of nitrogen, 28 g/mol.

The fuel burn rate (g/s) as denoted in Eq. (3) refers to the time required for complete combustion of the fuel in mixture with air and denotes the rate at which energy is released into the system. The higher the fuel burn rate, the more efficient the system and the higher the combustion temperature, as more energy is released.

(3) Fuel burnrate,FBR(g/s)=Mf/t(s)

where Mf is the mass of biomass consumed per experiment in g and *t* is the duration of the experiment in s.

The carbon mass flow rate (${\text{m}}_{\text{C}})$, carbon conversion efficiency (CCE) and modified combustion efficiency (MCE) were calculated by using Eqs. (4), (5), (6) respectively.

(4) $${\text{m}}_{\text{C}(\text{g}/\text{s})}=x_{\mathrm{fg}}\cdot {\text{M}}_{\text{C}}\cdot ([{\text{CO}}_{2}]+[\text{CO}]+[{\text{CH}}_{4}])$$

(5) $$\eta_{\text{CCE}}\text{,}\%=([{\text{M}}_{\text{C}}]/{\text{C}\%}_{\mathrm{fuel}}-{\text{C}\%}_{\mathrm{ash}})$$

(6) $$\text{MCE,}\%=[{\text{CO}}_{2}]/([{\text{CO}}_{2}]+[\text{CO}])$$

where $C{\%}_{\mathrm{fuel}}\;\text{and}\;C{\%}_{\mathrm{ash}}$ are the percentage of carbon in the fuel and ash respectively, and [M_C_] is the molecular weight of carbon, 12 g/mol.

The excess air ratio, λ was calculated based on the estimated stoichiometric air-to-fuel ratio molar fractions of the gases in the flue gas and fuel burn rate as expressed in Eq. (7).

(7) $$\lambda ={\text{AFR}}_{\mathrm{real}}/{\text{AFR}}_{\mathrm{stoic}}=(X_{i}\cdot \rho /\text{M}f\cdot {\text{AFR}}_{\mathrm{stoic}})$$

## 3 Results and discussion

### 3.1 Biomass characterization

The proximate and ultimate composition of the HF, WB and SF are summarized in Table 2. Particle size, bulk density, and calorific value of the fuels are also shown.

The results in Table 2 show that the volatile matter and ash content of the human faeces are 85.39 wt.% db and 14.56 wt.% db respectively. In the case of wood biomass, the volatile matter and ash contents are 13.5 points higher and 13 points lower respectively. In terms of elemental composition, the contents in C and H of the dry human faeces and wood biomass are similar. However, differences in the nitrogen and oxygen content are observed; particularly in the case of the percentage composition of oxygen, which is 23.10 ± 0.16 wt.% db in the case of HF and 43.04 ± 0.90 wt.% db in the case of WB. This is due to the slight differences in N and ash content of the fuels. The HHV values of both fuels are in the same order, with the human faeces having a slightly higher HHV of 24.73 ± 0.10 MJ/kg (around 13% higher than that of WB). Comparison between the human and simulant faeces shows that both samples present similar proximate compositions (with a percentage difference <2%). However, in the case of ultimate analysis, slight differences in the C and N content are observed, with a percentage difference of about 10% for C and >100% for N. Similar to wood biomass, the HHV of the simulant faeces is slightly lower than the human faeces, an acceptable percentage difference of ∼11%.

The main differences in fuel composition and their influence on combustion performance are discussed in Section 3.3. The composition results of the three samples are in agreement with composition data of similar samples published elsewhere [23]. Onabanjo et al. [23] showed that average composition from a set of 12 different human faeces sample was 51 ± 2 wt.% C, 7 ± 0 wt.% H, 4 ± 1 wt.% N, 21 ± 3 wt.% O and 17 ± 1 wt.% ash (dry basis). In addition, simulant faeces prepared following the same recipe used in this work had similar ultimate composition, i.e. 46 wt.% C, 8 wt.% H, 3 wt.% N, 30 wt.% O and 14 wt.% ash while the wood biomass, drawn from the same pool of sample had 49 wt.% C, 7 wt.% H, 0.2 wt.% N, 43 wt.% O and 0.7 wt.% ash. The close compositional characteristics of the simulant faeces to human faeces make it a suitable model fuel and the wood biomass serves as an appropriate reference biomass fuel.

### 3.2 Parametrisation

Series of experiments were conducted using simulant faeces and wood biomass to understand the combustion operating range of the test rig and to identify the optimum conditions for achieving complete combustion, under limited energy requirement for igniting the fuel. The results are presented in Sections 3.2.1–3.2.4.

#### 3.2.1 Influence of equivalence ratio and fuel ignition

**Fig. 2:**
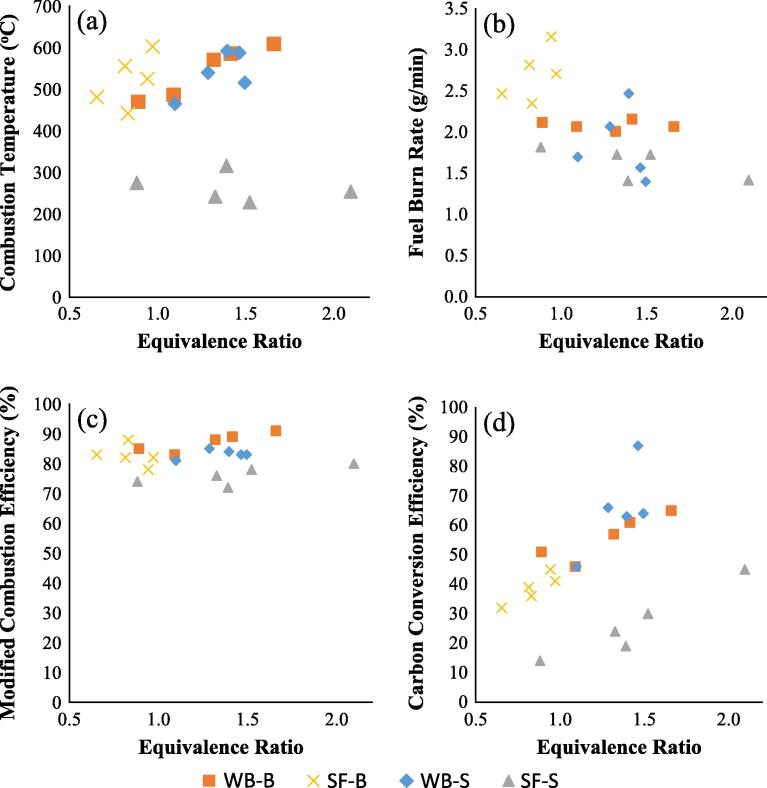
(a–d): Influence of equivalence ratio on: (a) combustion temperature, (b) fuel burn rate, (c) MCE, and (d) η_CCE_, as a function of fuel ignition mode (booster, B and standard, S) and varying fuel types (wood biomass, WB and simulant faeces, SF).

Fig. 2(a–d) presents the effect of equivalence ratio on fuel burn rate, combustion temperature, MCE and η_CCE_, considering two modes of ignition (standard and booster) for the use of simulant faeces and wood biomass.

Fig. 2a and b shows the significant influence of fuel ignition on the combustion processes of simulant faeces. Under standard ignition of the simulant faeces, the results in Fig. 2a show that combustion temperature varied between 228 °C and 316 °C across the different ERs. This range increased to 442 °C and 603 °C for the simulant faeces with booster ignition, a percentage difference of >40%. The fuel burn rate in Fig. 2b also increased from a range of 1.4–1.8 g/min to 2.4–3.2 g/min. In the case of the wood biomass, the combustion temperature was similar for both ignition types within the ER range of 1–1.5. The combustion temperature had a range of 465–592 °C for standard ignition, while 470–605 °C for booster ignition. The fuel burn rate had a range of 1.7–2.5 g/min for the case of the standard ignition of the wood biomass, and varied between 2.1 and 2.2 g/min for the booster ignition case.

Fig. 2c shows that the MCE was above 70% for all the fuel types, irrespective of the ignition type, which is a good indication that the test rig was operating within a combustion range. The combustion processes with booster ignition had higher MCE than their standard ignition counterparts. The use of booster ignition increased the MCE slightly from a range of 78–85% to 83–91%, a 9% increase at the least for the wood biomass, while it was 72–80% to 78–83% in the case of the simulant faeces, a 6% increase at least in MCE. The results in Fig. 2d also indicate a clear trend of the effect of ER on carbon conversion efficiency. For all the biomass materials and ignition types, there was more carbon conversion in the flue gas at higher ER. The η_CCE_ increased from the range of 14–45% to 32–45% for the simulant faeces, indicating the significant influence of the booster ignition. In the case of the wood pellet, the η_CCE_ had a range of 46–65% and 46–87% for booster and standard ignition respectively.

As suggested by Palmer [10] and Carvalho et al. [11], smouldering begins when the fuel reaches its ignition temperature; hence, the insignificant difference between booster and standard ignition for the wood biomass could be as a result of a fully satisfied fuel ignition process, such that the system becomes independent of the assisted heat supply. Wang et al. [25] showed that increased inflow of air enhanced flame propagation and this was attributed to improved mass transfer of oxygen to the surface of the combustible material at elevated temperatures. The diffusion of oxygen is said to increase the reaction rate and consequently the generation of heat. The improved interactions between the fuel and air therefore enables a higher heat of combustion. Nevertheless, a high air flow rate also increased the loss of heat because of convective heat transfer, and so a compromise is required. In some of the failed tests (results not shown), there was visible release of volatiles that turns into flames at elevated temperatures; however, the reaction rapidly extinguishes when the air flows (>16 L/min) are not favourable. In some of the instances, it was the result of excess air flows (>16 L/min) that affect flame stability, reduces the combustion temperature and extinguishes any visible flame. In other cases, it was as a result of insufficient air flows to propagate the flame and heat produced. In the instances of prolonged ignition period, typical of standard ignition, flame propagation was not achieved because convective heat transfer became dominant and the release of volatiles was not accompanied by high temperatures. Thus, controlled air supply is important for self-sustained ignition, propagation and combustion of the biomass samples; otherwise flame extinction occurs.

In this study, the optimum ER for the conversion of the simulant faeces with booster ignition was ∼0.94–0.97, which agrees with stoichiometric combustion. This ER corresponds to air supply rate of 14–18 L/min. The highest combustion temperature for the wood biomass was achieved within the ER of 1.3–1.4, also corresponding to air supply rate of 14–18 L/min. This form of low combustion temperature, slow progressing ignition and oxidation of the fuel, is classified as smouldering. While the process is occurring at higher temperatures in the wood biomass irrespective of the fuel ignition mode and in the booster ignition of the simulant faeces, it is characterised by a relative long reaction time of the fuel and having relatively low temperature in the simulant faeces (standard ignition).

#### 3.2.2 Influence of bed height

The influence of the bed height on the performance of the rig was tested with wood pellets of average size of 8 mm × 10 mm. Combustion was carried out at fixed air volumetric flow rate of 16 L/min at four bed heights; namely, 20, 30, 35 and 40 mm, which corresponds to 25, 50, 75 and 100 g of sample respectively (see Table 3). Wood biomass was chosen for the evaluation of bed height influence because of the limited effect of the mode of fuel ignition and the ease of ignition. The appropriate bed height for the combustion of human faeces was then extrapolated from these results.

As can be seen from Table 3, the combustion temperature increased when the bed height increased, ranging from 497 to 655 °C. Similar trend was observed in the case of fuel burn rate, which increased from 2.15 to 3.77 g/min when the bed height increased. The increment in combustion temperature and fuel burn rate with the bed height can be attributed to the larger amount of fuel in the reactor. Since more fuel is available for the combustion reaction, more heat of combustion is released into the system; hence, supporting the conversion of more fuel. Interestingly, the MCE and η_CCE_ showed optimum values of 86% and 72% respectively, corresponding to a bed height of 30 mm. At bed height of 40 mm, the reaction was observed to be operating near gasification (*results not shown*), due to the high amount of CO (up to 10%) in the gas relative to CO_2_. Hence, it is possible that part of the energy released from the exothermic combustion process is used to drive the endothermic processes of gasification of the solid residue. The bed height of 20 mm on the other hand exhibited excess air and reduced combustion bed temperature. Furthermore, it was observed that at above bed height of 30 mm, there was prolonged ignition period. This is because at bed height of 30 mm, the primary air enters into the combustion zone just above and directed at the solid fuel with uniform distribution of air, while at bed height of above 30 mm, the solid fuel is above the entry of the primary air and there is poor interaction between the air and solid fuel. The results obtained for combustion of wood biomass at varying bed height can have limited application in faeces combustion because of the significant amount of ash in the samples, as the ash can interfere with combustion processes, especially for continuous feeding processes. To this effect, a new combustor rig is under development to manage ash accumulation.

#### 3.2.3 Influence of fuel pellet size

Combustion of simulant faeces pellets of varying sizes was carried out to address the influence of this parameter on the performance of the rig and to establish adequate particle size for the faecal biomass. The simulant faeces sample was chosen for this analysis due to similar physical properties and chemical characterization to human faeces, as well as the homogeneous nature of the fuel, such that a definite pellet size can be prepared and test repeatability can be ensured. The experiments were conducted both at standard and booster ignition modes. The influence of fuel pellet size on combustion temperature, fuel burn rate, MCE and η_CCE_ are presented in Table 4. All these tests were conducted at constant air supply rate of 12 L/min, as this was the optimum condition for the standard ignition of the SF sample.

The results show that SS-2 (10 mm × 10 mm square shape pellets) with booster ignition had the highest performance among the fuel pellet sizes. Thus, a combustion temperature of 520 °C and a fuel burn rate of 2.91 g/min were achieved, which resulted in a MCE of 80% and η_CCE_ of 41%. The SS-4 (5 mm × 10 mm cylindrical pellets) with booster ignition had a similar performance, although with a lower combustion temperature of 484 °C, higher fuel burn rate of 3.31 g/min, and similar MCE of 80% and higher η_CCE_ of 48%. The rest of the fuel sizes (SS-1 and SS-3) with booster ignition had combustion temperature that was less than 300 °C. These samples also had extended reaction time of >20 min with values as low as 1.15 g/min for fuel burn rate, 72% for MCE and 18% for η_CCE_.

The simulant faeces (SS-2 and SS-4) with standard ignition had lower combustion temperature than their booster counterparts, with temperature difference of 179 °C for SS-2 and 168 °C for SS-4 pellet size cases. Consequently, the fuel burn rate, MCE and η_CCE_ had worse performance, with respective percentage difference of 11%, 1% and 2% for the SS-2 pellet size and 29%, 13% and 65% for the SS-4 pellet size. Comparison of the performance of SS-1 (0.5 mm × 0.5 mm) and SS-3 (20 mm × 20 mm) square-shaped pellets under both ignition modes points out that the booster ignition impeded the combustion process. SS-1 sample achieved a much lower combustion temperature with relatively long reaction time of up to 30 min when booster ignition was applied. This could be as a result of the inability of the preheated air to flow through the compact sample bed and the release of the volatiles being hindered. Similarly, the SS-3 fuel sample presented a worse performance with extended ignition period when booster ignition was applied. The results show that the reaction time of the fuel was up to 43 min, as compared to the 38 min in the case of the standard ignition. A lower combustion temperature was also reached with temperature difference of about 30 °C when booster ignition was applied. The fuel burn rate reduced by nearly 14% while MCE increased by 10%. This could be attributed to the relative large size of the fuel pellets that necessitate the passing of the preheated air through the combustion zone without proper interactions between the biomass material and hot air. This is evident with the relatively long reaction time of the fuel, low process temperatures and poor η_CCE_. Since, MCE is a relative percentage of CO_2_ to the mixture of CO_2_ and CO in the flue gas, the increased MCE in the booster ignition case can be attributed to the relatively long reaction time that permits more CO_2_ formation from CO in the product gas and O_2_ in excess air. The 10 mm × 10 mm square shaped pellets were the most suitable size for fuel conversion in the combustor test rig, as fuel size SS-3 pushes the reaction towards excess oxygen range and SS-1 encourages oxygen depletion.

#### 3.2.4 Influence of fuel moisture content

This set of experiments was carried out to investigate the minimum combustion temperature that is required for the combustion of human faeces with different contents of moisture. Onabanjo et al. [23] have described the moisture content of human faeces as “parasitic” when used as fuel; in other words, moisture content can cause a significant delay or failure to ignite the fuel and other subsequent conversion processes. This is because the energy that is released into the system is utilised in part or whole to evaporate the moisture in the sample. The moisture content can also compromise self-sustainability of the process. As shown in Table 5, simulant faeces with moisture content between 40 and 70 wt.% (wet basis) and minimum bed temperature between 400 and 600 °C were investigated. For each test, 50–75 g of wood biomass that can ensure the required minimum bed temperature was initially combusted in the reactor. At defined combustion bed temperature that corresponds to the point where charring starts, 50 g of the moist simulant faeces is then introduced into the reactor. At the end of each experiment, a test is classified as a success, if the moist faeces were completely burnt out to gas products and ash, and as a fail test, if the sample was partly converted or not all.

**Fig. 3:**
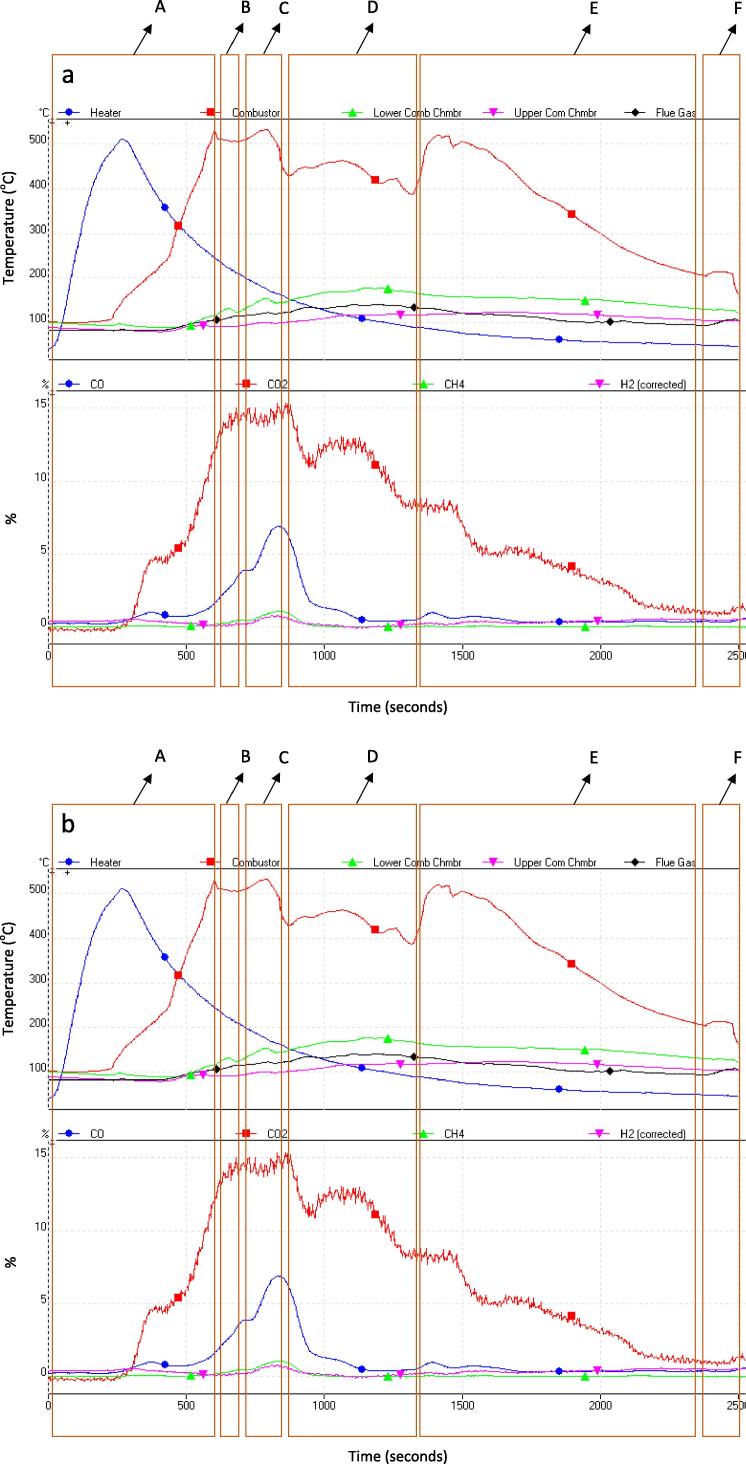
(a and b): Combustion of simulant faeces (60 wt.% moisture): (a) at combustion temperature of 600 °C, air volumetric flow rate of 14 L/min, (b) at combustion temperature of 500 °C, air volumetric flow rate of 14 L/min, adjusted to 10 L/min after ∼1500 s.

The process of fuel ignition and conversion of the simulant faeces with 65 wt.% moisture at 600 ± 10 °C and 500 ± 10 °C are shown in Fig. 3a and b respectively.

The process has been classified into the following stages: (A) wood biomass is ignited using the air igniter. The ignition airline is switched off once ignition was achieved (blue line). Combustion is allowed to take place until there is an observed charring stage, that corresponds to a decline in combustion bed temperature. (B) the fuel gate is opened and the sample is introduced into the fuel bed. This causes a slight decline in combustion bed temperature. (C) the fuel drop disturbs the bed and stokes the ash and this causes a sharp increase in combustion bed temperature. (D) the newly introduced moist fuel begins to dry and (E) propagates into flames after drying and volatilisation, if sufficient heat is still available in the system. Flame propagation is accompanied by increasing rise in combustion temperature (red line) while a continuous decline indicate the heat is continuously absorbed for drying, and (F) fuel gate is opened and unburnt sample is recovered, if available.

The results in Table 5 show that samples with as much as 65 wt.% of moisture in the fuel can be processed provided the combustion bed temperature is at least 500 °C or higher at optimum air-to-fuel ratio. This minimum bed temperature is critical if a self-sustained combustion process should be achieved. In this study, 50 g of moist biomass was introduced into the reactor as a batch and this means that the heat recovered from the combustion of wood biomass is only used for drying the biomass due to the high amount of moisture in the sample. Thus, there would be need for fuel re-ignition if new set of samples were to be introduced. In ideal systems, this process will require an automated and controlled feed-in system and rig design that would allow sufficient time for the fuel to dry and progressive addition of the moist fuel, without overwhelming the on-going combustion process. Table 5 also shows that a different air flow rate (10 L/min) was applied for the moist samples (>55 wt.%) at minimum combustion temperature of 500 °C and for the moist sample (>40 wt.%) at minimum combustion temperature of 400 °C. This is because the air flow rate of 16 L/min was observed to rapidly reduce the combustion temperature while the lower air flow rate of 10 L/min allowed the moist fuel to completely dry and gradually combust, even at very low temperatures. These observations indicate the critical importance of controlled air supply and minimum temperature of the bed. In the experiments with success, the moist samples were completely converted to ash with <1% of residual carbon.

### 3.3 Human faeces and combustion performance

Based on the Bristol Stool Chart (BSC), human faeces can be categorised by physical appearance into 7 types of stool. The stool types vary from ‘Type 1’ stool, classified as ‘separate hard lumps, like nuts’ to ‘Type 7’ stool, listed as ‘watery, no solid pieces’, as shown in Table 6 [26]. The 2–4 type stools were commonly found among the samples collected.

**Fig. 4:**
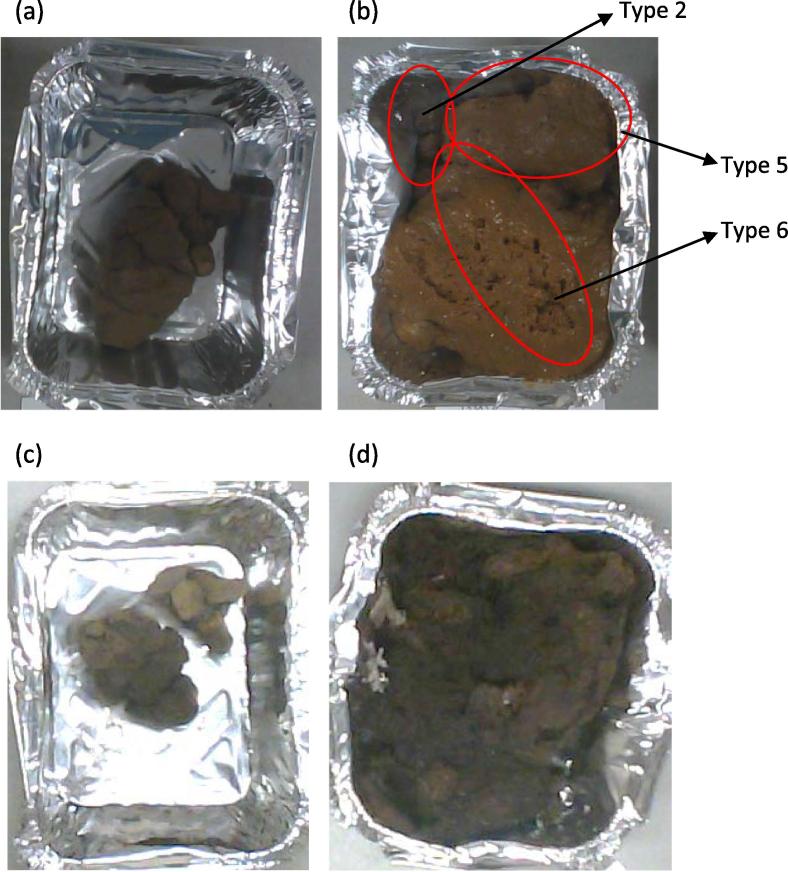
(a–d): Human Faeces: (a) Type 2 fresh sample (as-received basis, prior to drying. (b) Mixture of Type 2, 5 and 6 fresh sample (as-received basis, prior to drying). (c) Type 2 sample (after drying). (d) Mixture of Type 2, 5 and 6 sample (after drying).

The various stool types also exhibited different moisture content levels, with the Type 1 having the least moisture content (∼50%) and Type 7 having the most (>80%). However, some of the samples in the study had definite forms as described in the BSC (Fig. 4a) while other samples were a mixture, including Type 2 and Types 4–6 stool characteristics, in some instances (Fig. 4b). Because of the heterogeneous dispersion of moisture content across each sample and the mixture of stool types even within a given sample, the various classes of stools are observed to exhibit different drying profiles (Fig. 4c and d) and the overall moisture content level in the sample did not defer significantly. For instance, the sample in Fig. 4a and b had ∼65 and ∼76 wt.% moisture, although the Type 6 section of the mixed sample ∼86 wt.% moisture.

The type 1–2 stools were observed to dry completely as a lump with minimal ‘shrinkage’, whereas the type 5–7 stools dried typically as sheets or flakes of solids with high ‘shrinkage’, that is a large reduction in volume of the sample in relation to the original volume. This could because the type 1–2 stools have a visible compact structure, hold less visible air spaces, and relatively low fuel moisture. The type 6–7 stools are not so compact and can hold high volume of gases that disperses during the dry stages. Hence, sample homogeneity was an important aspect of the faeces combustion tests.

To minimise fuel variations, all the collected samples were mixed together to obtain a relatively uniform consistency and distribution of undigested foods such as vegetables and grains. Nevertheless, a definite pellet size could not be achieved due to mass shrinkage of the sample during drying and non-uniform in drying characteristics. As such, the samples were bulk dried at minimal oven temperature of 45 °C and crushed to an estimated mean pellet size of 10 mm. Based on the experimental outcomes in Sections 3.2.1–3.2.4, the combustion analyses of dry human faeces were conducted at air flow rate of 10–18 L/min using booster ignition. For consistency and appropriate comparison, 50 g of the dry sample that corresponds to a bed height of 30 mm was introduced into the reactor. The result findings are indicated in Table 7.

The results in Table 7 show that the combustion temperature for the dry human faeces increased from 431 to 558 °C while the fuel burn rate increased from 1.53 to 2.30 g/min, with increasing air supply rate, corresponding to ER of 0.86–1.12. These results show that the combustion processes for the human faeces were close to stoichiometric combustion, an ideal condition where the carbon and hydrogen in the faeces mixes with sufficient air and reaches a maximum temperature and complete conversion to H_2_O and CO_2_. The optimum combustion temperature and fuel burn rate were obtained at the highest air supply rate of 18 L/min, due to increased oxidation of the fuel. While the highest burn rate was achieved at air flow rate of 18 L/min, the highest MCE and η_CCE_ were obtained at air flow rate of 10 L/min, a range of 77–89% for MCE and 53–82% for η_CCE_. The improved combustion efficiencies at low air supply rates can be attributed to the long residence time of the fuel (>30 min) that provides more time for the sample to be completely burnt out to CO_2_ and H_2_O.

Among the different operating conditions, fuel ignition sequence and fuel characteristics had a significant influence on the combustion processes of the dry human faeces. Fuel ignition is a critical parameter that affects the amount of heat released into the system, as it has a direct influence on combustion temperature and fuel burn rate. Monhol and Martins, [20] exposed faeces to heat influx of 30 kW/m^2^ from a radiant cone heater at elevated temperatures of 570 °C and achieved a combustion temperature of about 885 °C. Their studies showed that the ignition temperature of human faeces is about 220–375 °C and exhibits a heterogeneous behaviour. In the case of an ‘heterogeneous’ fuel ignition, there is direct interactions of oxygen and the organic matter on the surface of the fuel; however, ‘homogenous’ ignition occurs in the gas surrounding the fuel [11]. In this study, the combustion of faeces can be described to exhibit smouldering ignition with complex heterogeneous reactions that transit into flame propagation. This observation holds for faeces with ‘booster’ ignition, because the heat flux from the air igniter increases the temperature of the fuel directly and enhances the thermal decomposition, such that drying, pyrolysis and release of gaseous volatiles are fastened for oxidation to occur. In the case of the standard ignition, the fuel is gradually heated and the volatiles are released, causing smouldering ignition to be dominant without flame propagation, resulting in a low combustion temperature, as described in Section 3.2.1. Similar to the simulant faeces, the maximum combustion temperature achieved under standard ignition of the dry faeces was at most 300 °C at optimum air flow rate of 16 L/min (*results not shown*). Since, minimal energy requirement is one of the design considerations for a self-sustained energy conversion system for the NMT, the input power is estimated at ∼1.2 kW for booster ignition and ∼2.9 kW for standard ignition. During the booster ignition of the dry faeces, the air igniter was operated at heater temperature between 620 °C and 627 °C for 5–6 min. This includes the time required for the heater to reach >600 °C and the period in which the suction fan is used to draw ambient air across the heated igniter surface until fuel ignition is achieved in the combustion zone.

Fuel characteristics is another critical parameter that influences the performance of the combustion process. In Section 3.1, it was mentioned that there are slight differences in the proximate compositions of WB and HF. The volatile matter content of the dry WB was up to 13% higher than those of HF with insignificant amount of ash. These slight differences had influence on combustion temperature (<600 °C) as higher values were reached with WB. Volatile matter is readily given off during combustion after drying of the sample, and can contribute to spontaneous combustion of the fuel, as it encourages fuel oxidation [27]. Ash on the other hand is largely inert and do not contribute to combustion temperature, but rather absorbs heat during combustion. Kaymakci and Didari [27] showed that ash can reduce the spontaneous ignition of coal and attributed this to certain components. Hence, a high composition of ash in the samples can reduce the temperature that could have been given off by the process. In smouldering combustion, ash can act as a porous medium to aid the flow of oxidant and increases the surface area for the fuel and air to interact. It is also suggested that ash act as thermal insulator to conserve the heat generated and to prevent heat loss into the environment [8]. Both phenomena were observed in the conversion of HF and SF. The accumulation of ash on the combustion bed appears to serve as a heat sink thereby allowing combustible material to have sufficient time to be heated and to follow through with the processes of combustion. However, excessive accumulation of ash appears to disrupt the combustion process. In these instances, the inlet of the air igniter and the primary air flow were found to have a blockage. Since the test rig has a downdraft configuration, the build-up of ash also disrupts the passage of air and product gas, thereby limiting the reaction considerably. Therefore, the design and development of a fuel conversion system for application in sanitary systems require an efficient ash control system.

Another aspect of fuel composition is the elemental constituents of C, H, O, N. Rose et al. [1] showed that human faeces contain large amount of undigested cellulose, vegetable fibres and pentosane that varies with dietary intake and depends on the fraction of absorbed soluble carbohydrate. Here, readily available carbohydrate such as starch and sugars increases faecal energy and conversely for unavailable carbohydrates. A high fibre content in the faeces from undigested plant matter also increased the calorific value of the fuel. In this study, the fixed carbon of the sample was in insignificant amount, suggesting readily available carbon in the fuel that corresponds to higher HHV of the fuel. Since, HHV is the gross amount of heat produced on complete combustion of a given mass of fuel, the relatively high HHV of the faeces can be related to the elemental composition of the fuels, mainly hydrogen:carbon weight (H/C) and oxygen:carbon weight (O/C) ratios. The HF have a slightly higher H/C, while lower O/C than the WB, respective values of 0.15 and 0.47 for HF and 0.14 and 0.88 for WB. The slightly higher hydrogen per carbon contributes to a lower oxidation state of the fuel and more energy release. The reduced oxygen per carbon also promote more oxidation of the fuel, hence more CO_2_ formation, as shown by the MCE (<80%) for the HF samples. Although, the dry HF sample had a relatively high HHV than WB, the dry HF was less an ignitable material than wood biomass and this can be related to the different volatile and ash contents. Finally, the HF sample had the highest N content; but this is irrelevant for this study, as N does not take part in combustion except at very high temperatures (>1800 °C), and this is outside the range of this study.

Beyond the analyses on the dry human faeces, attempt was made to combust moist (as received basis) human faeces at optimum air flow rate of 16 L/min and minimum bed temperature of 600 °C, using Type 2 (62 wt.% moisture) and Type 6 (88 wt.% moisture) stool types. All the six attempts had no success rate, largely due to the non-definite form of the moist faeces. Although, the Type 2 faeces was able to hold a form, it was held bound by mucous as the sample was introduced into the reactor. The heat generated from the wood biomass combustion was however only sufficient to partly dry the moist faeces, without fuel ignition. Further work will therefore aim at creating a homogenous faeces pellet size.

The work presented in this study is expected to influence the design, development and optimisation of an appropriate energy conversion system for the NMT. The toilet is intended to safely treat human waste onsite under limited energy requirement and achieve self-sustained operation with no external power supply. Hence, the temperature between 431 °C and 558 °C obtained at air flow rate of 10–18 L/min and the minimum combustion temperature of about 500 °C for combusting dewatered, partly dried faeces suggests the need for a well-insulated system that can ensure minimal heat loss into the environment. Also, of high importance is the need to ensure the safety of the users, thus particular attention is being given to the choice of material for the design of the new combustor to ensure it retains heat, unlike current combustor rig that dissipates heat to the surroundings. The reported fuel burn rates of 1.53–2.30 g/min at air flow rates of 10–18 L/min imply that dry human faeces from about 55–82 persons can be treated every day, assuming an average faeces generation rate of 40 g/cap/day (dry basis). Rose et al. [1] indicated a wide range of faeces generation rate of 51–796 g/cap/day (as received basis), with a median value of 128 g/cap/day. In low- and high-income countries, respective median fresh faeces generation rates of 250 and 126 g/cap/day were reported. Total solids were in the range of 11–34% with a median value of 25%. Thus, at the upper limit of faeces generation rate, the fuel burn rate still corresponds to the daily dry faecal treatment of 11–16 persons, and this meets one of the objectives of the NMT to treat faeces of single-household use (equivalent to 10 people). For optimal continuous operation and low fuel burn rate, equivalent to 10 people, a miniature combustion system is being developed. An automated ash control system is also being developed to ensure that ash accumulation does not hinder the faeces conversion processes. A drying chamber with multiple capabilities as a pelletiser is planned to ensure extended operation and continuous feed and drying of samples. Because energy recovery will depend on drier characteristics, fuel composition, mainly moisture content of the incoming samples, and the efficiency of the heat recovery system, further investigation will aim to establish the appropriate trade-off limits for drying and energy recovery under the consideration of different stool types, moisture content and drying characteristics.

## 4 Conclusions

Experimental investigation of faeces combustion was carried out using a bench-scale downdraft combustor test rig. Air flow rate, fuel pellet size, bed height, and fuel ignition mode were varied in order to establish the combustion operating range of the test rig and the optimum condition required for using converting the faecal biomass. Wood biomass and simulant faeces samples were also used to preliminary evaluate the performance of the equipment. Performance evaluation was carried out on the basis of combustion temperature, fuel burn rate, MCE and carbon conversion efficiency. The experimental results show that dry human faeces have a higher energy content than wood biomass on a dry basis. Fuel ignition had a significant influence on the maximum combustion temperature in the combustor test rig. Here, the exposure of elevated temperatures of up to 600 °C for a very short duration of about 60 s (booster ignition) was sufficient to ignite the fuel and push the reaction towards flame combustion. Fuel burn rate of 1.5–2.3 g/min was achieved at air flow rate of 10–18 L/min. Increased air flow rate improved combustion bed temperature, however reduced the reaction time and consequently the carbon conversion and combustion efficiencies of the fuel. Further work will be required at creating a homogenous faeces pellet size and to establish the appropriate trade-off limits for drying and energy recovery under the consideration of different stool types, moisture content and drying characteristics. This can improve the drying processes of faeces combustion significantly and can ensure the successful development of a self-sustained energy conversion and recovery systems for the NMT and similar sanitary solutions.

## Figures and Tables

**Table 1:** Recipe for simulant faeces [22].

Ingredients	Dry weight (g/kg)
Baker’s yeast	72.8
Peanut oil	38.8
Miso paste	24.3
Propylene glycol	24.3
Cellulose powder	12.4
Psyllium husk powder	24.3
Calcium phosphate	25.0
Water^a^	778.1

a Water was added based on the required moisture content.

**Table 2:** Chemical and physical properties of the different biomass feedstock.

Samples	Bulk density kg/m^3^	Particle size^b^ (mm)	Proximate analysis (wt.% dry basis)			Ultimate analysis (wt.% dry basis)				HHV (MJ/kg)
Volatile matter	Fixed carbon	Ash content	C	H	N	O^a^
WB	600 ± 18	8 × 10	98.70 ± 0.00	0.22 ± 0.00	1.04 ± 0.00	48.96 ± 0.80	6.88 ± 0.11	0.08 ± 0.00	43.04 ± 0.90	21.54 ± 0.38
SF	626 ± 5	5 × 10	86.77 ± 0.00	0.08 ± 0.00	13.15 ± 0.00	44.85 ± 0.10	7.24 ± 0.03	2.52 ± 0.01	32.24 ± 0.10	22.36 ± 0.06
HF	277 ± 45	10 × 10	85.39 ± 0.00	0.05 ± 0.00	14.56 ± 0.00	49.41 ± 0.11	7.62 ± 0.04	5.31 ± 0.02	23.10 ± 0.16	24.73 ± 0.10

HF – Human Faeces, WB – Wood Biomass, SF – Simulant Faeces; Oxygen.

a 100 – (wt.% of C, H, N and ash).

b Average size reported.

**Table 3:** Influence of bed height for wood biomass combustion.

Bed height (mm)	Peak combustion temp (°C)	Fuel burn rate (g/min)	MCE (%)	η_CCE_ (%)	Duration of experiment (min)
40	655	3.77	71	66	26.5
35	631	2.64	82	68	28.4
30	607	2.44	86	72	20.5
20	497	2.15	80	56	11.6

Normal ignition; Air Flow Rate – 16 L/min; Wood Biomass.

**Table 4:** Influence of fuel pellet size and ignition for simulant faeces combustion.

Sample	Pellet size (mm × mm)	Ignition type	Peak combustion temp (°C)	Fuel burn rate (g/min)	MCE (%)	η_CCE_ (%)	Duration of experiment (min)
SS-1	5 × 5, square shape	B	215	1.56	72	22	32.0
SS-2	10 × 10 square shape	B	520	2.91	80	41	17.2
SS-3	20 × 20 square shape	B	203	1.15	80	24	43.5
SS-4	5 × 10 cylindrical shape	B	484	3.31	80	48	15.1
SS-1	5 × 5, square shape	S	318	2.44	67	28	20.5
SS-2	10 × 10 square shape	S	341	2.59	79	40	19.3
SS-3	20 × 20 square shape	S	243	1.31	72	27	38.2
SS-4	5 × 10 cylindrical shape	S	316	2.36	70	17	21.2

B-Booster; S-Standard.

**Table 5:** Minimum temperature required for ignition and complete conversion of moist simulant faeces samples.

Moisture content (wt.%)	Combustor bed temperature		
600° ± 10 °C	500 ± 10 °C	400 ± 10 °C
70%	x	x	x
65%	√−F_air_ = 14 L/min	√F_air_ = 16 L/min^a^	x
60%	√F_air_ = 16 L/min	√F_air_ = 16 L/min^a^	x
55%	–	√F_air_ = 16 L/min	x
50%	–	–	x
45%	–	–	x
40%	–	–	√ F_air_ = 10 L/min^a^

√Success at air flow of 16 L/min; (a) air flow rate of 14 L/min; (b) air flow rate of 16 L/min; (c) air flow rate of 10 L/min.

a Adjusted to 10 L/min at low combustion temperature below 400 °C; – analysis not conducted based on previous success rate; x – failed test.

**Table 6:** Bristol stool chart [26] with broad classification of moisture content from study.

Type 1		“separate hard lumps, like nuts”	∼50%
Type 2		“sausage-shaped but lumpy”	50–65%
Type 3		“like a sausage but with cracks on the surface”
Type 4		“like a sausage or snake smooth and soft”	65–80%
Type 5		“soft blob with clear cut edges”
Type 6		“fluffy pieces with ragged edges, a mushy stool”	>80%
Type 7		“watery, no solid pieces”

**Table 7:** Combustion performance at varying air supply rate.

Sample	Air volumetric flow rate (L/min)	Ignition type	Peak comb. temp (°C)	Duration of experiment (min)	Fuel burn rate (g/min)	MCE (%)	η_CCE_ (%)
HF	18	B	558	21.8	2.30	77	55
HF	16	B	542	25.1	1.99	83	63
HF	14	B	547	24.7	2.02	87	68
HF	12	B	431	26.1	1.91	87	57
HF	10	B	435	32.7	1.53	89	82
